# Binding of Tau-derived peptide-fused GFP to plant microtubules in *Arabidopsis thaliana*

**DOI:** 10.1371/journal.pone.0286421

**Published:** 2023-06-02

**Authors:** Hiroshi Inaba, Kazusato Oikawa, Kazuya Ishikawa, Yutaka Kodama, Kazunori Matsuura, Keiji Numata

**Affiliations:** 1 Department of Chemistry and Biotechnology, Graduate School of Engineering, Tottori University, Tottori, Japan; 2 Centre for Research on Green Sustainable Chemistry, Tottori University, Tottori, Japan; 3 Department of Material Chemistry, Graduate School of Engineering, Kyoto University, Kyoto, Japan; 4 Center for Bioscience Research and Education, Utsunomiya University, Tochigi, Japan; 5 Biomacromolecules Research Team, RIKEN Center for Sustainable Resource Science, Wako, Saitama, Japan; University of Rome, ITALY

## Abstract

Studies on how exogenous molecules modulate properties of plant microtubules, such as their stability, structure, and dynamics, are important for understanding and modulating microtubule functions in plants. We have developed a Tau-derived peptide (TP) that binds to microtubules and modulates their properties by binding of TP-conjugated molecules *in vitro*. However, there was no investigation of TPs on microtubules *in planta*. Here, we generated transgenic *Arabidopsis thaliana* plants stably expressing TP-fused superfolder GFP (sfGFP-TP) and explored the binding properties and effects of sfGFP-TP on plant microtubules. Our results indicate that the expressed sfGFP-TP binds to the plant microtubules without inhibiting plant growth. A transgenic line strongly expressing sfGFP-TP produced thick fibrous structures that were stable under conditions where microtubules normally depolymerize. This study generates a new tool for analyzing and modulating plant microtubules.

## 1. Introduction

Microtubules are tubular cytoskeletons composed of tubulin dimers that serve fundamental and crucial roles in the biological processes of various eukaryotes including animals, plants, fungi, and protists. Microtubules play diverse roles in cell morphology, intracellular transport, cell growth and division, and in the formation of the mitotic and cytokinetic apparatus [1–7]. Because of the importance of plant microtubules, small-molecule drugs such as oryzalin, propyzamide, and taxol have been used to regulate their dynamics, organization, and stability, and have also been developed as potential herbicides [2, 3]. In nature, microtubule-associated proteins (MAPs) modulate the structures and functions of microtubules, including their stability, polymerization, bending, and bundling, and their ability to deliver cargo [8, 9]. In addition to MAPs that bind to the outer surface of microtubules, microtubule inner proteins (MIPs) that bind to the inner surface of microtubules have recently been discovered [10, 11]. The binding of MIPs to the inner surface of microtubules possibly increases the mechanical rigidity and stability of microtubule structures. Fluorescent protein-fused MAPs have been widely used for visualization of microtubules in plants [12, 13]. Pioneering works by Cyr et al. showed the binding of microtubule-binding domain of the human MAP-4 fused with green fluorescent protein (GFP) to microtubules in plants [14–16]. Other MAPs such as MAP65-1, MAP65-2, MAP65-4, and MAP70 are also used for the visualization [17–21]. Interestingly, the expression of the fluorescent protein-fused MAPs may affect the properties of microtubules such as stability and bundling [12, 13, 15, 18, 20]. These findings suggest that the development of exogenous proteins that bind to plant microtubules could be a new approach to modulate microtubule function in plants.

**Fig 1:**
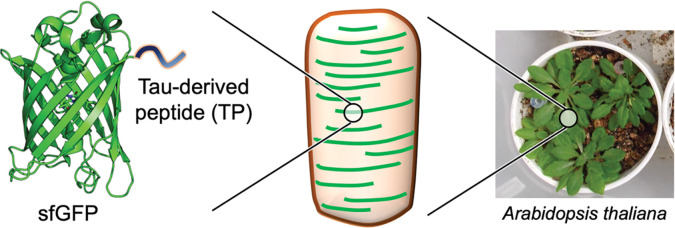
Schematic illustration of binding of Tau-derived peptide (TP)-fused superfolder GFP (sfGFP-TP) to microtubules of *Arabidopsis thaliana*.

Previously, we developed a Tau-derived peptide, TP (CGGGKKHVPGGGSVQIVYKPVDL) as a binding motif for the inner surface of microtubules [22]. The design of TP was based on a repeat domain of the Tau protein, which is conserved from nematodes to humans and is known to bind to the inner pocket of microtubules [23]. TP was derived from R2-interrepeat (298–312) of human 4R-Tau. When TP is pre-incubated with tubulin followed by the polymerization of the TP-tubulin complex, it binds to the inner surface of microtubules. Using this method, TP has been used to encapsulate various nano-sized materials such as superfolder GFP (sfGFP) [24], Azami-Green protein (a tetrameric fluorescent protein) [25], magnetic cobalt–platinum nanoparticles [26], and gold nanoparticles [22, 27] in microtubules. Encapsulation of these nanomaterials changed the properties of microtubules *in vitro* [11]. For example, binding of TP-fused sfGFP (sfGFP-TP) to the inner surface of microtubules increased the contour length, rigidity, and velocity on kinesin-coated substrates, as well as microtubule stability [24]. In that case, binding of both TP and the sfGFP scaffold were possibly important for the stabilization of microtubules. In contrast, sfGFP-TP binds to the outer surface of microtubules when it is incubated with pre-formed microtubules [24]. In addition, TP can bind to microtubules in human hepatoma HepG2 cells without cytotoxicity [28, 29]. These properties suggest that binding of exogenous proteins to microtubules *via* TP has great potential to modulate the structures and functions of plant microtubules. To date, however, there have been no attempts to introduce TP-conjugated molecules into plants. There are potential applications of TP-fused fluorescent proteins in plants because they can be stably expressed, and their inherent fluorescence makes them visible under a microscope. Compared with the transient effects of microtubule-targeted drugs, stably expressed sfGFP-TP in plants may permanently alter the structure and functions of plant microtubules and make it easier to visualize the altered structures due to its fluorescence. Here, we analyzed the binding of sfGFP-TP to microtubules in the model plant *Arabidopsis thaliana* and its stabilizing effect (Fig 1).

## 2. Material and methods

### 2.1. Generation of transgenic plants expressing sfGFP-TP

A synthetic DNA fragment encoding sfGFP was cloned into pDONR207 using the BP reaction following the manufacturer’s protocol (Thermo Fisher Scientific, Waltham, MA, USA). A linear pDONR207-sfGFP-TP sequence fragment was amplified by inverse PCR using pDONR207-sfGFP as the template with the following primers: 5′-CATGTTCCAGGAGGTGGTTCAGTCCAGATCGTTTATAAACCTGTGGATCTTTGAGACCCAGCTTTCTTG-3′ and 5′-GACTGAACCACCTCCTGGAACATGTTTTTTACCGCCACCAGAACCTCCACCAGTGATCCCGGCGGCGGT-3′. The resulting fragment was circularized using the In-Fusion HD Cloning Kit (TaKaRa, Otsu, Japan) to generate a pDONR207-sfGFP-TP plasmid. The cloned sfGFP-TP fragment was recombined into the pGWB602 binary vector using the LR reaction [30] following to manufacturer’s protocol (Thermo Fisher Scientific). Transgenic *A*. *thaliana* plants stably expressing sfGFP-TP were produced by *Agrobacterium tumefaciens* (GV3101)-mediated transformation using the floral-dip method [31]. The plants were grown at 23°C on 0.8% agar plates containing half-strength Murashige and Skoog medium with 1% w/v sucrose (pH 5.7) in an incubator (Nihonika, Osaka, Japan) under a 16-light/8-h dark cycle at 100 μmol photons m^-2^ s^-1^ after vernalization. The leaf length was directly measured from the plant and the primary root length was measured by pulling the roots out of the agar plates, laying them on a flat agar medium, and stretching them in a straight line. The confocal laser scanning microscopy (CLSM) analyses were conducted on 2–3-week-old plants. To examine the relationship between sfGFP-TP and microtubules, transgenic lines stably expressing sfGFP-TP and mCherry-fused tubulin (mCherry-TUB6) [32] were crossed and F3 homozygous lines were generated.

**Fig 2:**
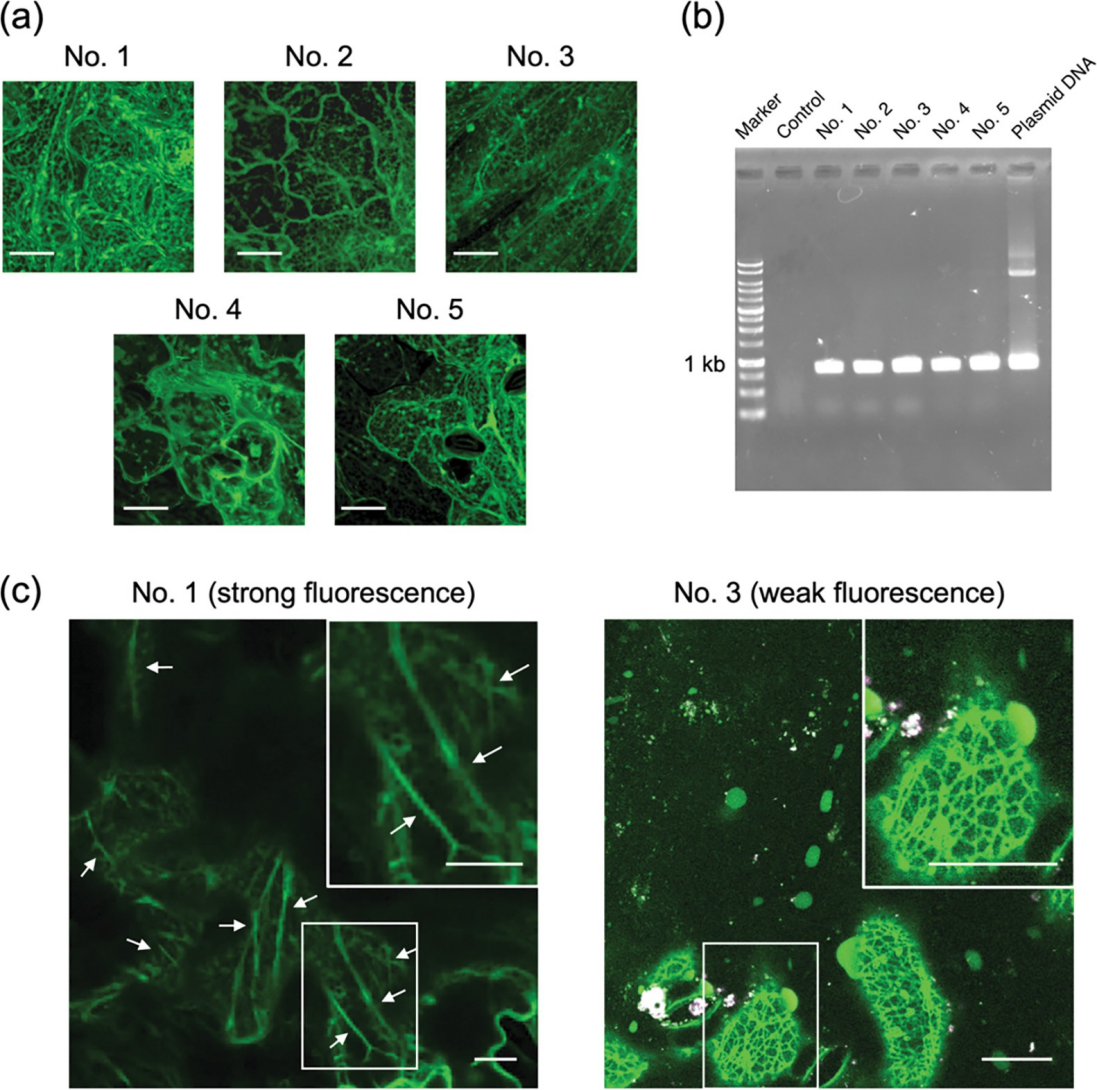
(a) CLSM images of selected sfGFP-TP-expressing *Arabidopsis* lines (No. 1–5), with green fluorescence of sfGFP-TP visible. (b) Agarose gel electrophoresis of PCR products of extracted genomic DNA corresponding to *sfGFP-TP* gene from each transgenic plant. Control is from a representative wild-type plant. (c) Images showing detailed structures of leaf epidermis of No. 1 line (strong fluorescence) and No. 3 line (weak fluorescence). White arrows indicate representative thick fibers. Inset: expanded images. Scale bars: 20 μm for (a) and (c). See S1, S2 Movies for cross-sectional scans acquired at different focal planes. The fluorescence intensity of sfGFP-TP in all lines was modified to be equal using Brightness/Contrast in Image J. Original CLSM images of No. 1 and No. 3 lines are shown in S3 Fig in S1 Data.

The insertion of the *sfGFP-TP* gene into the *A*. *thaliana* genome was confirmed by PCR using the following primer set: Forward, 5′-ACGTAAACGGCCACAAGT-3′, and reverse, 5′-AACGATCTGGACTGAACC-3′. Genomic DNA was extracted from each transgenic *A*. *thaliana* line using plant DNAzol™ Reagent (Thermo Fisher Scientific) and purified by washing with ethanol. The PCR analyses were performed using a C1000 Touch™ Thermal Cycler (BIO-RAD, Hercules, CA, USA) with PrimeSTAR^®^ GXL DNA Polymerase (TaKaRa) following the manufacturer’s protocol. An amplified DNA (approx. 1 kb) corresponded to a part of *sfGFP-TP* in the transgenic lines (Fig 2B). The sfGFP-TP lines were classified according to the strength of the fluorescence signal (No. 1–5) detected using a Leica M165 FC fluorescence stereomicroscope (Leica Microsystems, Tokyo, Japan). Two lines, No. 1 and No. 3, were selected for subsequent experiments.

### 2.2. Imaging analysis

Light-adapted adult rosette leaves were deaerated and placed on a glass coverslip in pure water for CLSM analyses. Etiolated-hypocotyl and -root epidermis after 3–5-days-old-dark-adaptatuion were placed on a glass coverslip in pure water for CLSM analyses. The images of *A*. *thaliana* plants expressing sfGFP-TP and mCherry-TUB6 were captured under a CLSM (Zeiss LSM880, Carl Zeiss, Jena, Germany) with a 63× oil immersion objective (Plan-Apochromat 40× and 63×/1.4 Oil DIC M27; Carl Zeiss). An argon laser was used for excitation of sfGFP-TP (488 nm) and mCherry-TUB6 (561 nm). Emission wavelengths at 507–525 nm and 580–630 nm were used for detection of sfGFP-TP and mCherry-TUB6 fluorescence, respectively. Z-axis sections were taken every 0.35 or 0.5 μm from the surface of epidermis cells, with a total thickness of at least 10 μm, to include the whole epidermal cell. The images were modified to be the same fluorescence intensity among sfGFP-TP lines using Brightness/Contrast tool in Fiji (ImageJ, NIH public domain). The images were stacked to be clear using Z projection in Fiji for epidermal cells of roots.

### 2.3. Analysis of effects of depolymerization conditions

The deaerated-leaf sections were immersed in a solution containing 10, 25, 50, or 100 μM oryzalin (Sigma-Aldrich, St Louis, MO, USA) or 0.1, 1.0, or 10 μM Latrunculin B (Wako Pure Chemical Co. Ltd., Osaka, Japan) for 3 h under white light conditions. Alternatively, leaf sections were kept at 4°C for 1 h. Images were captured as Z-sections by CLSM. The number of individual fibers visualized by sfGFP-TP in a cell was manually counted by using Cell Counter tool in Fiji (ImageJ, NIH public domain) (S5 Fig in S1 Data). At least 11 cells from three different sections were quantified.

## 3. Results

### 3.1. Preparation of the transgenic plants stably expressing sfGFP-TP

**Fig 3:**
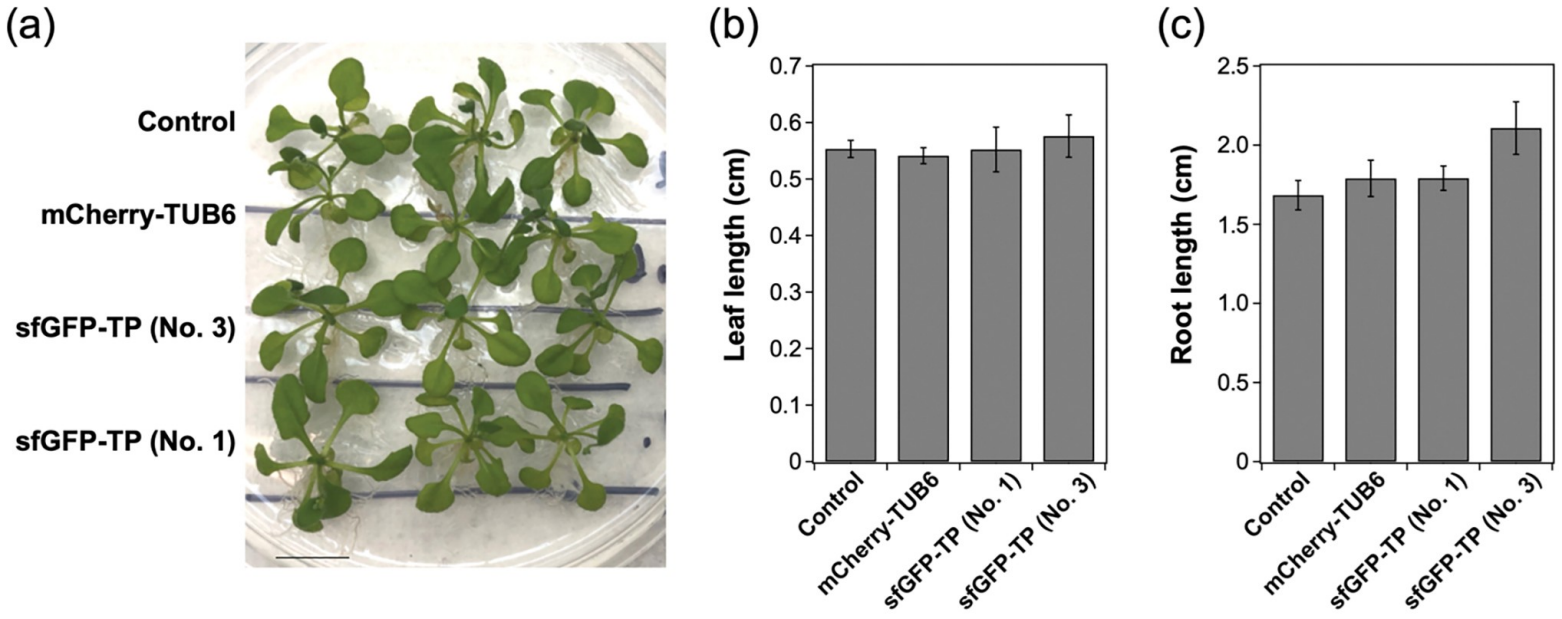
Plant growth test of sfGFP-TP-expressing *Arabidopsis thaliana* (No 1; strongest sfGFP-TP fluorescence, No. 3; weakest sfGFP-TP fluorescence). (a) Plants grown on agar plate under white light at 100 μmol photons m^-2^ s^-1^ for 4 weeks. Scale bar: 1 cm. The length of (b) leaf and (c) main root estimated from the plants are represented as the mean ± standard error of the mean (*N* = 6).

**Fig 4:**
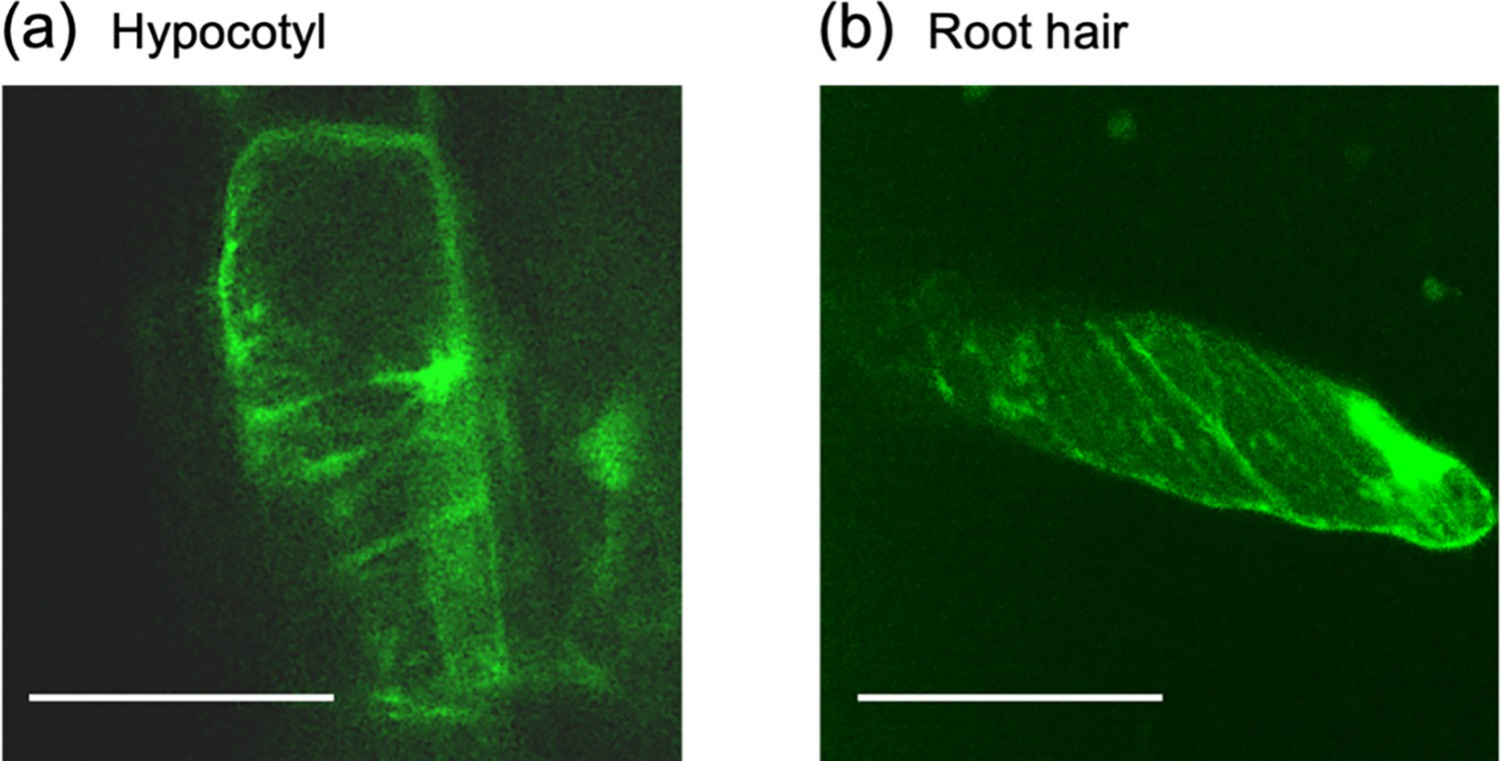
CLSM images of epidermal cells of (a) hypocotyl and (b) root hair of the sfGFP-TP-expressing *Arabidopsis* line (No. 3) with green fluorescence of sfGFP-TP visible. The images were stacked using Z projection in Fiji for (b). Scale bars: 50 μm.

In this work, we designed the whole sequence of sfGFP-TP consisting of sfGFP, which shows strong fluorescence even in plants [33, 34], joined with a GGGS linker to the TP sequence for expression in plants (S1 Fig in S1 Data). Compared with the previous reconstructed system [24], this system with the whole sfGFP-TP sequence is suitable for stable expression in plants and binding to plant microtubules without the requirement for translocation across the barriers of plant cell walls and plasma membranes. Transgenic plants stably expressing sfGFP-TP were generated by *Agrobacterium tumefaciens-*mediated transformation with the floral-dip method [31]. The expression of sfGFP-TP in the plants was confirmed by observing sfGFP fluorescence under a fluorescence stereomicroscope (S2 Fig in S1 Data). From the primary transformants expressing sfGFP-TP, we selected the five independent transgenic plants (No. 1–5) showing the highest fluorescence intensity in the leaf cells, as observed by CLSM (Fig 2A). The genomic insertion of the *sfGFP-TP* gene was confirmed by polymerase chain reaction (PCR) analysis of the extracted genomic DNA from each transgenic plant. The presence of an amplified DNA (around 1 kb), corresponding to a part of *sfGFP-TP* in the transgenic lines (Fig 2B), confirmed the successful insertion of the *sfGFP-TP* gene. In this study, two of the five lines, No. 1 (strongest sfGFP-TP fluorescence) and No. 3 (weakest sfGFP-TP fluorescence), were selected for subsequent experiments (S3 Fig in S1 Data). In both lines, expression of sfGFP-TP had no apparent effects on plant growth (Fig 3). Thick fibrous structures, which could be cytosolic strands (Fig 2C, left, white arrows, S1 Movie) were present in cells of the No. 1 line, whereas thin fibrous structures were present in cells of the No. 3 line (Fig 2C, right, S2 Movie). Since the structures and morphologies in the No. 3 line were similar to those reported previously for plant microtubules [35], we evaluated the binding of sfGFP-TP in the No. 3 line to epidermal cells of hypocotyl and root hair, where cortical microtubules are abundant in an orderly pattern. The transverse microtubule arrays were observed in the sfGFP-TP channel (Fig 4), indicating the binding of sfGFP-TP to the plant microtubules. In contrast, the thick fibers in the No. 1 line were different from typical plant microtubules observed in the No. 3 line. Also, the strong fluorescence of sfGFP-TP in the No. 1 line was observed in regions that appeared to be either cytoplasmic strands or cortical endoplasmic reticulum (Fig 2C, S3 Fig in S1 Data, and S1 Movie), suggesting that a portion of the overexpressed sfGFP-TP is localized in these areas.

### 3.2. Co-visualization of sfGFP-TP and mCherry-TUB6-labeled microtubules

**Fig 5:**
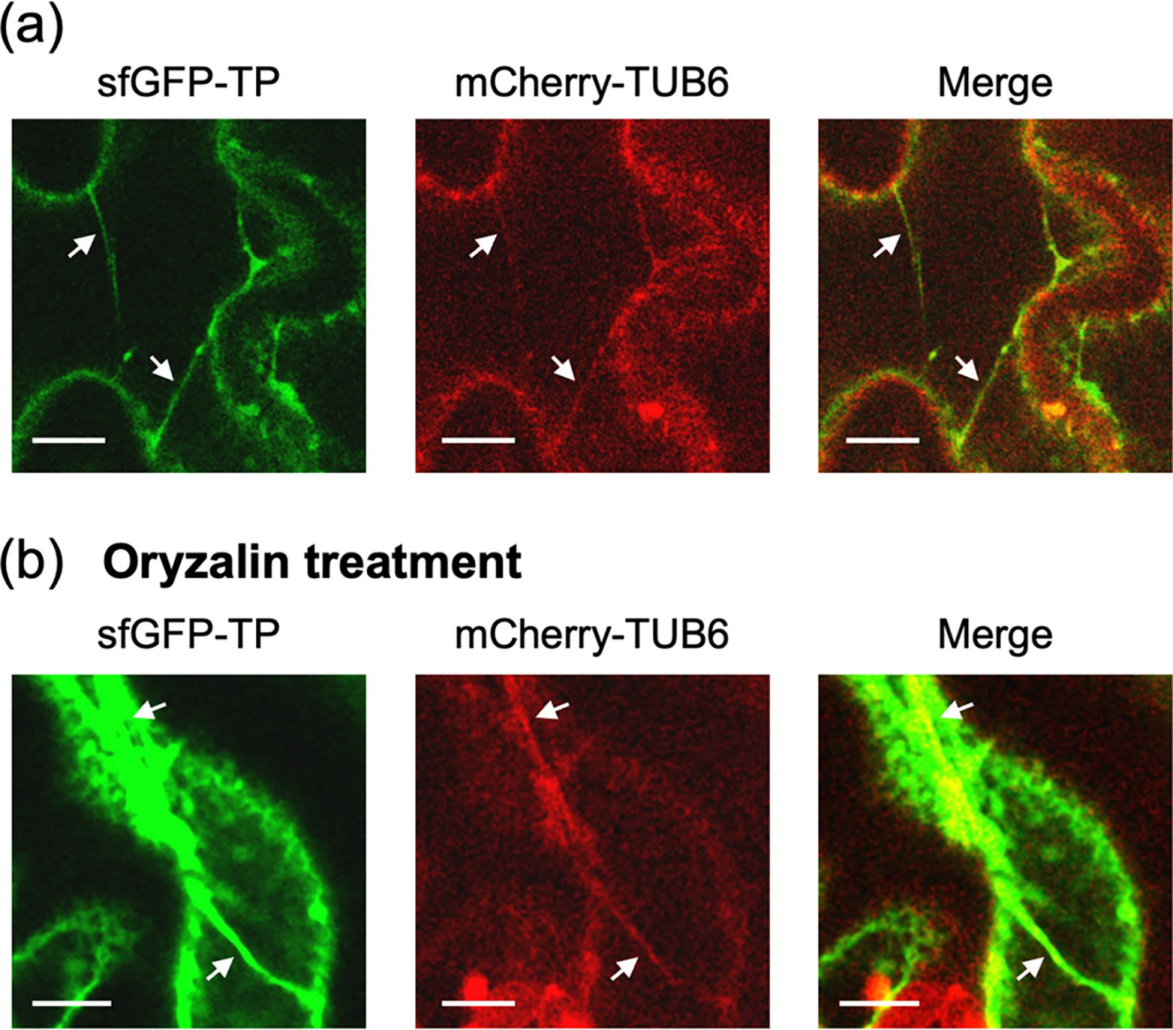
Colocalization assay of sfGFP-TP and mCherry-fused tubulin (mCherry-TUB6). CLSM images of homozygous plants expressing sfGFP-TP (No. 1 line) and mCherry-TUB6 with (a) no treatment and (b) treatment with 50 μM oryzalin for 3 h. White arrows indicate representative thick fibers. Scale bars: 10 μm for (a) and (b). See S3, S4 Movies for cross-sectional scans acquired at different focal planes.

To co-visualize sfGFP-TP and microtubules, the No. 1 line (strongest sfGFP-TP fluorescence) was crossed with a line expressing mCherry-fused tubulin (mCherry-TUB6) [32] to generate a homozygous line. Colocalization of sfGFP-TP and mCherry-TUB6 was observed in the thick fibrous structures (Fig 5A, S3 Movie), confirming the binding of sfGFP-TP to microtubules in the thick fibers. However, a partial lack of colocalization of sfGFP-TP and mCherry-TUB6 suggested the localization of a certain fraction of sfGFP-TP in either cytoplasmic strands or cortical endoplasmic reticulum. Next, the effects of a microtubule-depolymerizing drug (oryzalin) [32, 36] on the thick fibers were evaluated. Even after treatment with 50 μM oryzalin for 3 h, the thick fibrous structures were still partially intact and the colocalization of sfGFP and mCherry fluorescence signals was maintained (Fig 5B, S4 Movie). When oryzalin was treated to the line expressing only mCherry-TUB6, the thick fibers were not observed (S4 Fig in S1 Data). These results indicate that the thick fibers bound to sfGFP-TP were stable against depolymerization conditions of microtubules.

### 3.3. Characterizations of the fiber structures with oryzalin and latrunculin B

**Fig 6:**
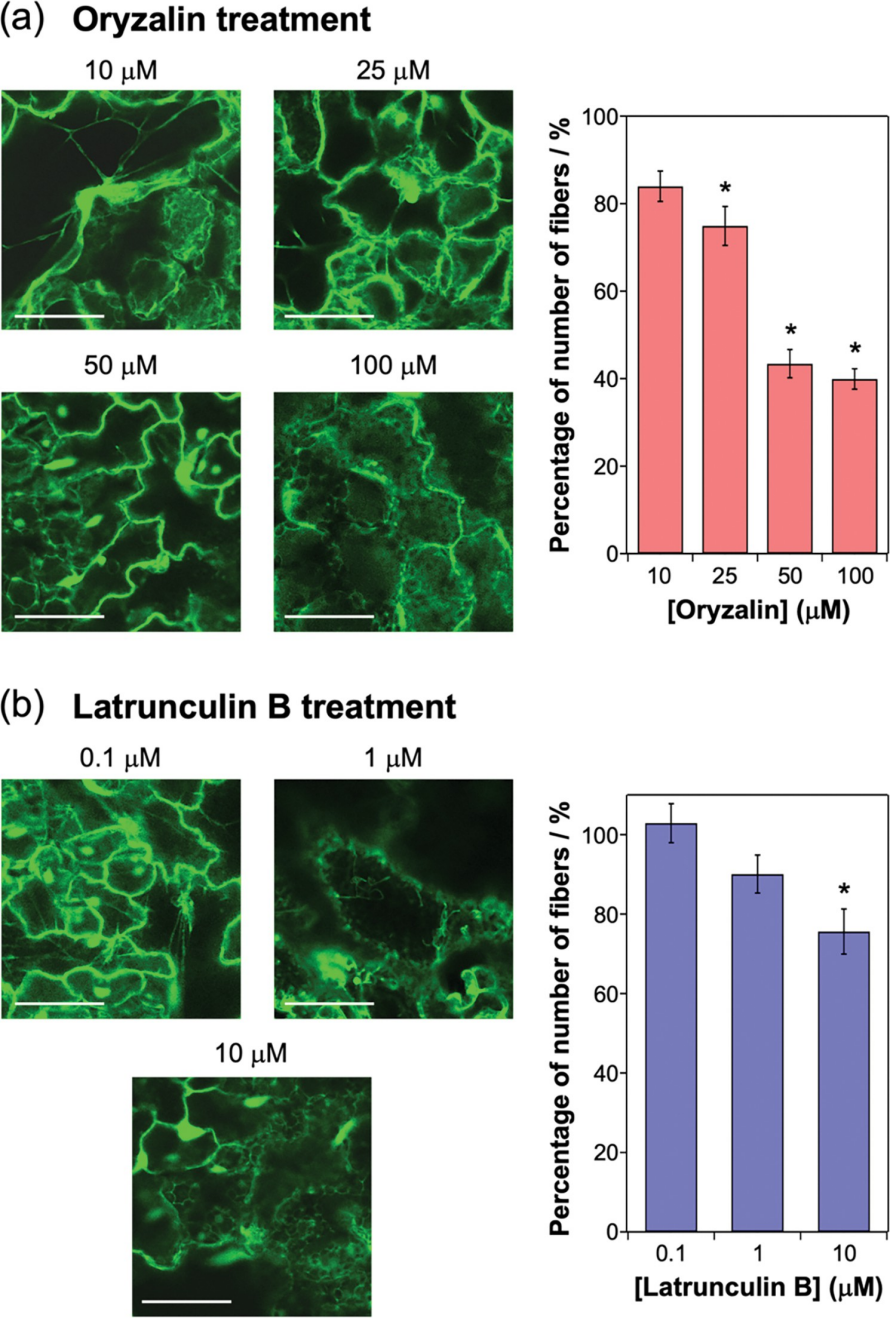
Effects of inhibitors of (a) microtubules and (b) actin filaments on sfGFP-TP-incorporated fibrous structures in the No. 1 line. CLSM images of leaf cells of plants treated with (a) oryzalin or (b) latrunculin B for 3 h (left). Scale bars: 50 μm for (a) and (b). Percentage of number of fibers per cell compared with the control cells (no oryzalin or latrunculin B treatment) (right). The number of fibers counted from three CLSM images are represented as mean ± standard error of the mean (*N* = 15 for (a) and *N* = 11 for (b)). *P < 0.01 compared with number of fibers in the control, as determined by two-tailed Student’s t-test.

**Fig 7:**
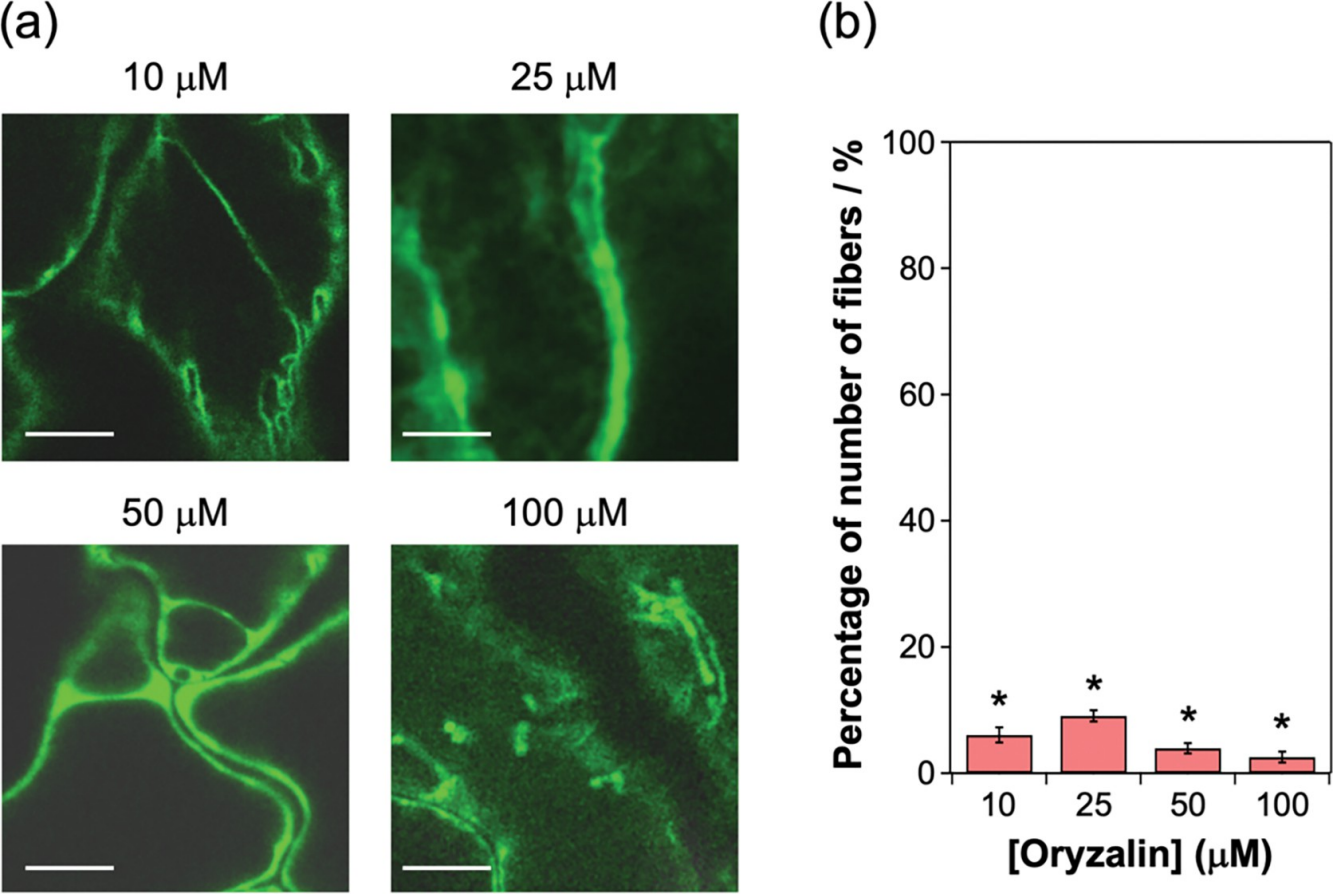
Effects of inhibitors of microtubules on the sfGFP-TP-incorporated fibrous structures in the sfGFP-TP-expressing *Arabidopsis* line (No. 3). (a) CLSM images of leaf cells of the plant treated with oryzalin for 3 h. Scale bars: 10 μm. (b) Percentage of number of fibers per cell compared with the control cells (no oryzalin treatment). The number of fibers counted from three CLSM images is represented as mean ± standard error of the mean (*N* = 15). *P < 0.01 compared with number of fibers in the control cells, as determined by two-tailed Student’s t-test.

Next, the structures of the thick fibers in the No. 1 line (strongest sfGFP-TP fluorescence) were further assessed by analyzing the concentration-dependent effects of depolymerizing drugs on microtubules and actin filaments (other parts of the cytoskeleton). Although microtubules and actin filaments fulfill many functions independently, they also interact, and this crosstalk is important for plant growth [36–39]. For instance, microtubules and actin filaments form dynamic associations during interphase in plant cells [36]. Thus, the effects of both a microtubule-depolymerizing chemical (oryzalin) [32, 36] and an actin filament-disrupting chemical (latrunculin B) [32, 36] on the fibers in the No. 1 line were evaluated. Typically, low concentrations of oryzalin (no more than 250 nM) were used to partially depolymerize cortical microtubules [40, 41]. In this study, higher concentrations of oryzalin (10–100 μM) were used to evaluate the stability of microtubules. To evaluate the effects of the chemicals, the number of fibers per cell was counted and compared with the control cells (no treatment of the chemicals) (S5 Fig in S1 Data). Oryzalin treatment decreased the number of fibers in a concentration-dependent manner (Fig 6A), indicating that the fibers were composed of microtubules, as shown in Fig 5. Although treatment with 20 μM oryzalin is known to depolymerize microtubules in plants [32, 36], the thick fibers remained largely intact at this concentration (Fig 6A). In contrast, the number of thin fibrous structures in the No. 3 line (weakest sfGFP-TP fluorescence) was significantly decreased by treatment with 10 μM oryzalin (Fig 7). Thus, the thick fibers in the No. 1 line were considerably more stable against oryzalin-induced depolymerization compared with the general microtubules and thin fibers in the No. 3 line. The thick fibers in the No. 1 line were not significantly lost after treatment with 1 μM latrunculin B, a concentration normally used to disrupt actin filaments (Fig 6B) [32, 36]. These results indicate that the fibers were bundled structures mainly formed by the interaction between microtubules.

## 4. Discussion

Two transgenic lines expressing sfGFP-TP (No. 1 and 3) in this study showed different localization of sfGFP-TP. The No. 3 line (weakest sfGFP-TP fluorescence) showed thin fibrous structures as similar to typical plant microtubules (Figs 2C and 4), showing the binding of sfGFP-TP to plant microtubules. In contrast, the No. 1 line (strongest sfGFP-TP fluorescence) showed thick fibers that were different from typical plant microtubules. In addition, strong fluorescence of sfGFP-TP was observed to different regions, possibly cytoplasmic strands or cortical endoplasmic reticulum (Figs 2C, 5A, S3 Fig in S1 Data, and S1 and S3 Movies). It is possible that a portion of the overexpressed sfGFP-TP in the No. 1 line is localized in these areas, whereas the moderate expression in the No. 3 line showed that sfGFP-TP was mainly bound to microtubules.

In the No. 1 line, the number of fibers were decreased by the oryzalin treatment but not dramatically decreased by the latrunculin B treatment (Fig 6), indicating that the fibers were bundled structures mainly formed by the interaction between microtubules. Since high concentration (10 μM) of latrunculin B partially disrupted the bundled structures (Fig 6B), it is also possible that the bundled structures contain actin filaments. In plants, this kind of bundled structures composed of microtubules [20] and microtubules/actin filaments [42] is generated by the binding of MAPs to microtubules. Also, the natural Tau protein can crosslink microtubules and actin filaments *in vitro* [43]. Because sfGFP-TP binds the outer surface of preformed microtubules [24], it is possible that the large amount of sfGFP-TP expressed in the No. 1 line mediates similar physical interactions between microtubules and/or microtubules and actin filaments to form stable bundles, like natural MAPs and Tau. Also, it is possible that disruption of actin filaments by latrunculin B treatment altered the organization of microtubules as reported in preprophase bands [39]. In further research, detailed analyses of the sfGFP-TP-incorporated fibers and the binding sites of sfGFP-TP should be conducted to determine their exact composition.

In the previous study, we used purified TP conjugates for binding to microtubules. The generation of genetically transformed plants expressing sfGFP-TP that binds to microtubules *in vivo* is an important advance for future applications. The stable expression of sfGFP-TP enables long-term stabilization of microtubules and their visualization. Compared with conventional studies that have investigated the short-term effects of microtubule-targeted small molecules, longer-term analyses of materials stably expressing sfGFP-TP can explore the effects of microtubule structures on plants without inhibiting plant growth (Fig 3).

In conclusion, we constructed an expression system of sfGFP-TP in the model plant *A*. *thaliana* and showed that the binding of sfGFP-TP to microtubule-based fibers. As discussed above, the No. 3 line showed the binding of sfGFP-TP to microtubules like MAPs [12, 13], whereas the No. 1 line generated thick fibers. By controlling the expression levels of sfGFP-TP, this system will be a new *in vivo* tool not only for imaging of plant microtubules, but also for elucidation and modulation of plant microtubules, which are still largely unknown compared with those of microtubules in other eukaryotes.

## Supporting information

S1 Data (PDF)

S1 Movie Cross sectional scans of the sfGFP-TP-expressing plant line (No. 1) in Fig 2C acquired at different focal planes. (MOV)

S2 Movie Cross sectional scans of the sfGFP-TP-expressing plant line (No. 3) in Fig 2C acquired at different focal planes. (MOV)

S3 Movie Cross sectional scans of the plant line (No. 1) expressing sfGFP-TP and mCherry-TUB6 without any treatment in Fig 5A acquired at different focal planes. (MOV)

S4 Movie Cross sectional scans of the plant line (No. 1) expressing sfGFP-TP and mCherry-TUB6 treated with 50 μM oryzalin for 3 h in Fig 5B acquired at different focal planes. (MOV)

S1 Raw images (PDF)
