# Supplementary material for: Binding of Tau-derived peptide-fused GFP to plant microtubules in Arabidopsis thaliana
Source: PLoS One. 2023 Jun 2;18(6):e0286421. doi: 10.1371/journal.pone.0286421 (PMC10237443; doi:10.1371/journal.pone.0286421)
Supplement: S1 Data — (PDF) [file pone.0286421.s001.pdf]

Supplementary Figures

MVSKGEELFTGVVPILVELDGDVNGHKFSVRGEGEGDATNGKLTLKFICTTGKLPVPWPTLVTTLTY
GVQCFSRYPDHMKQHDFFKSAMPEGYVQERTISFKDDGTYKTRAEVKFEGDTLVNRIELKGIDFKED
GNILGHKLEYNFNSHNVYITADKQKNGIKANFKIRHNVEDGSVQLADHYQQNTPIGDGPVLLPDNHY
LSTQSVLSKDPNEKRDHMVLLEFVTAAGITGGGSGGGKKHVPGGGSVQIVYKPVDL

Supplementary Fig 1. Amino acid sequence of sfGFP-TP used in this study. The green indicates sfGFP and blue indicates TP.

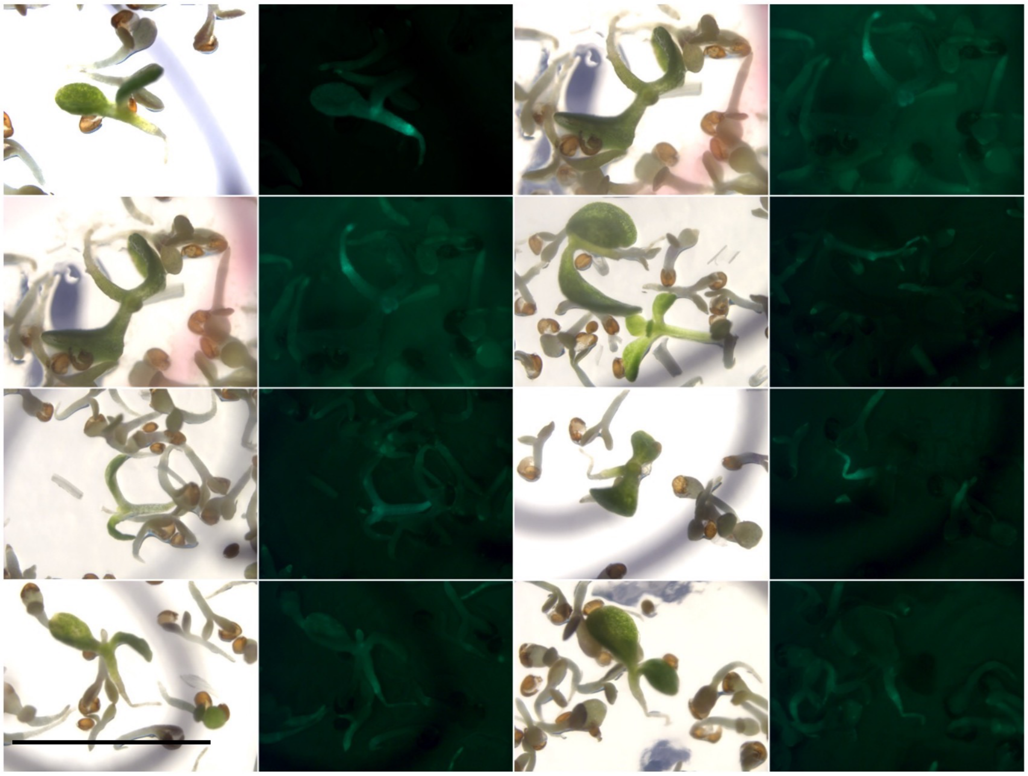

Supplementary Fig 2. Typical fluorescence stereomicroscope images of primary selected transformants of sfGFP-TP-expressing plants. Scale bar: 0.5 cm.

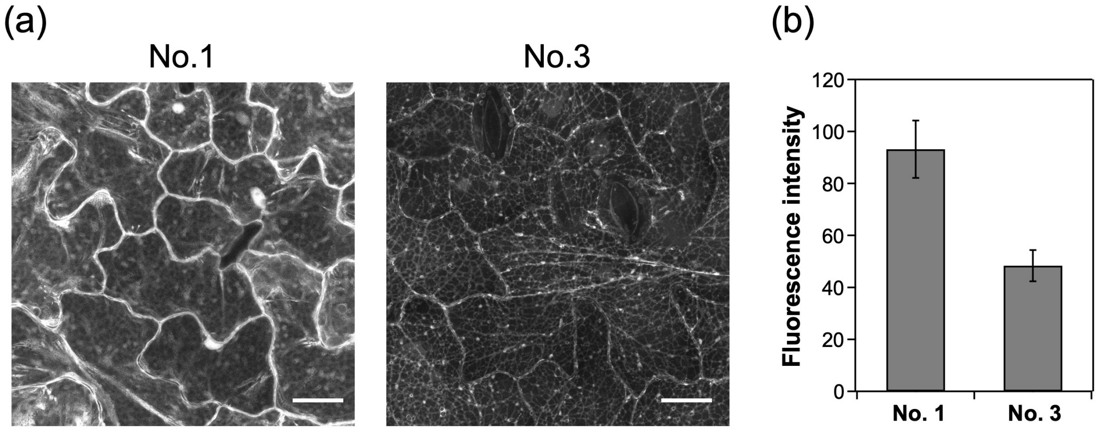

Supplementary Fig 3. Comparison of fluorescence of sfGFP-TP in the transgenic plants. (a) Representative CLSM images of leaf cells of the sfGFP-TP-expressing *Arabidopsis* lines (No. 1 and No.3). Scale bars: 20 μm. (b) The fluorescence intensity of sfGFP-TP in No. 1 and No. 3 lines is represented as the mean ± standard error of the mean ($N = 6$).

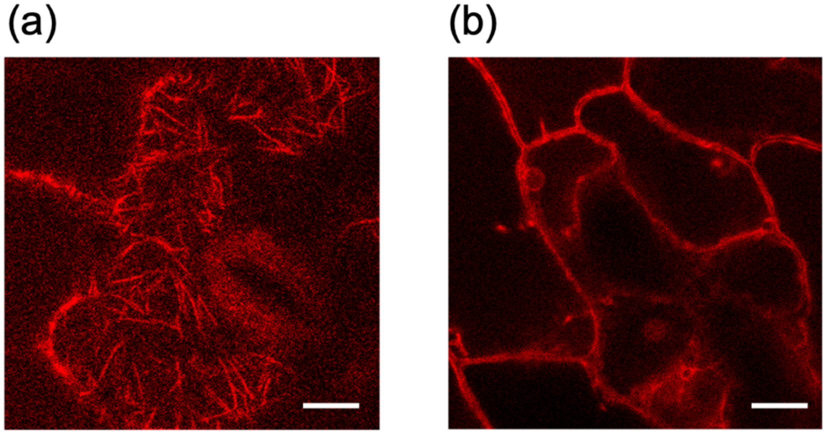

Supplementary Fig 4. CLSM image of leaf cells of the mCherry-TUB6-expressing *Arabidopsis* lines with (a) no treatment and (b) treatment with 100 μM oryzalin for 3 h. Scale bars: 10 μm.

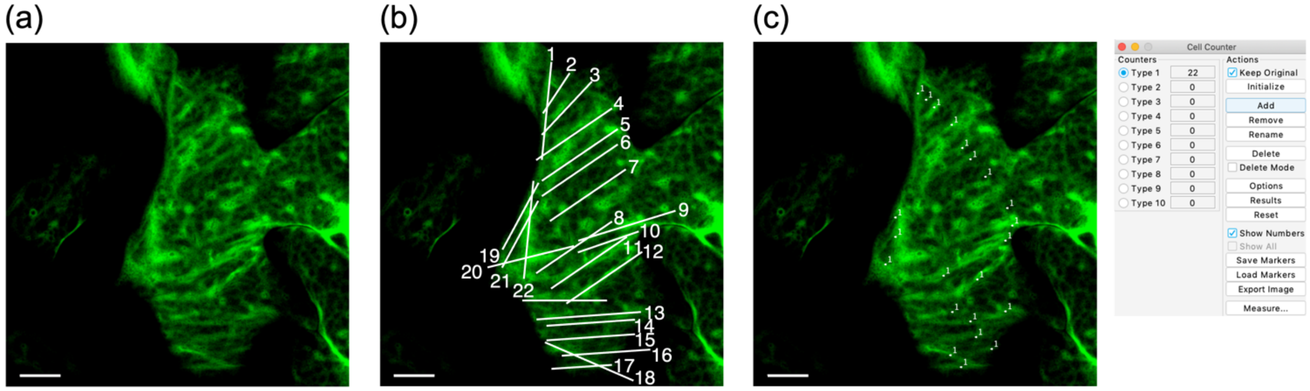

Supplementary Fig 5. Counting the number of fibers from CLSM images. (a) The original image. (b) Selection and (c) counting of fibers. Scale bars: 10 μm. The image sections were taken at intervals of 0.35 μm from the cell surface to a depth of 10 μm. The fibers at the top of an epidermal cell were carefully selected (white line in (b)) and the endoplasmic reticulum-like structures seen in the deeper regions of the cell were excluded from the counting process. The number of the fibers visualized with sfGFP-TP was manually counted using Cell Counter Plugin in Fiji as shown in (c). Each fiber was tagged with ‘1’ and total number was shown in the Cell Counter window.
