# Supplementary material for: Binding of Tau-derived peptide-fused GFP to plant microtubules in Arabidopsis thaliana
Source: PLoS One. 2023 Jun 2;18(6):e0286421. doi: 10.1371/journal.pone.0286421 (PMC10237443; doi:10.1371/journal.pone.0286421)

Original Image

Fig 2b is an electrophoresed agarose gel image. PCR products were electrophoresed onto an agarose gel and stained with ethidium bromide. ExcelBand 1KB (0.25-10kb) DNA Ladder (DM3100, SMOBIO Technology, Inc.) was used as a molecular weight size marker. Gel image was captured using WSE-6100H LuminoGraph I with ImageSaver6 software (ATTO Co., Ltd., Tokyo, Japan).

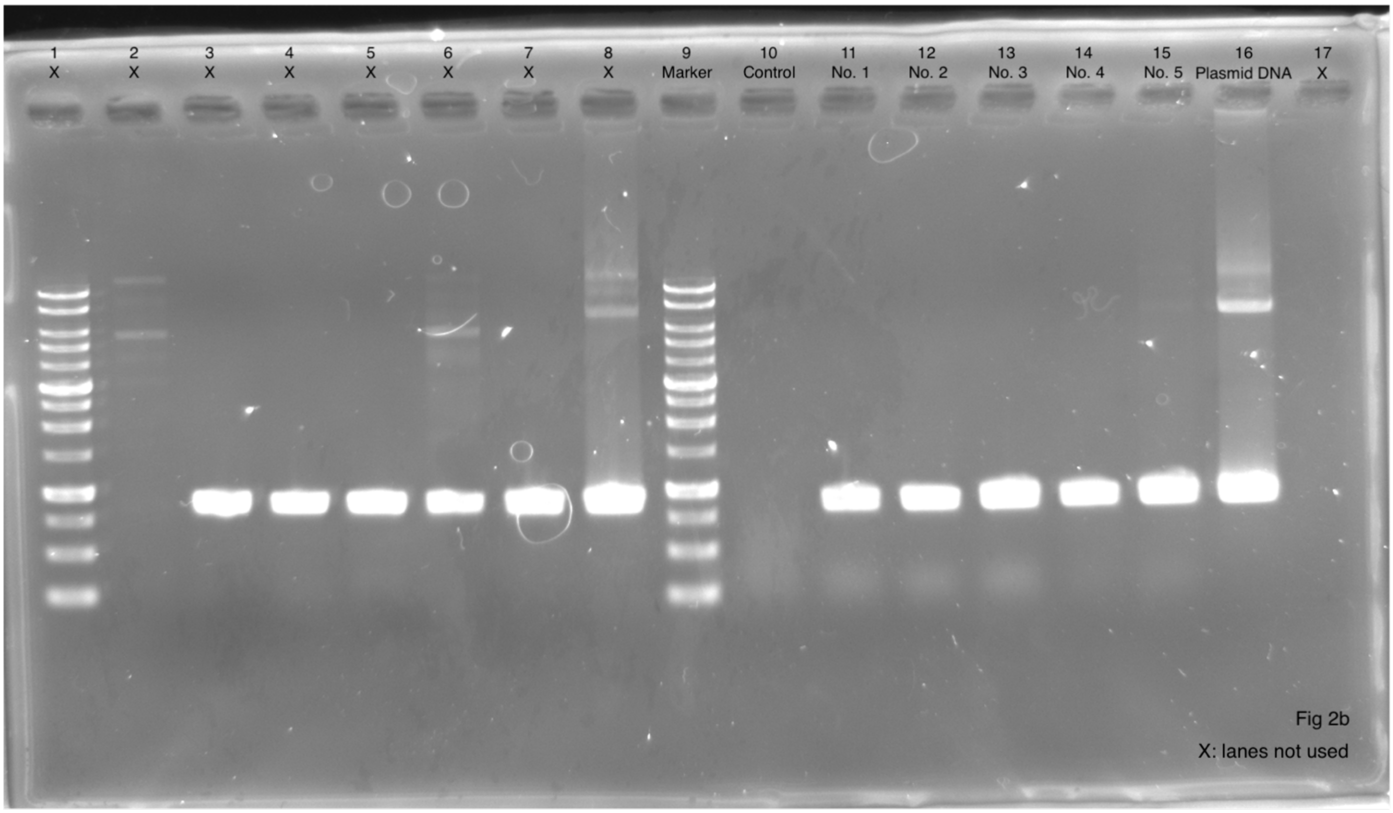

Supplement: S1 Raw images — (PDF) [file pone.0286421.s006.pdf]
